# Dopamine Transporter Imaging for Frontotemporal Lobar Degeneration With Motor Neuron Disease

**DOI:** 10.3389/fnins.2022.755211

**Published:** 2022-02-25

**Authors:** Ryota Kobayashi, Shinobu Kawakatsu, Makoto Ohba, Daichi Morioka, Masafumi Kanoto, Koichi Otani

**Affiliations:** ^1^Department of Psychiatry, Yamagata University School of Medicine, Yamagata, Japan; ^2^Department of Neuropsychiatry, Aizu Medical Center, Fukushima Medical University, Aizuwakamatsu, Japan; ^3^Department of Radiology, Yamagata University Hospital, Yamagata, Japan; ^4^Department of Diagnostic Radiology, Yamagata University School of Medicine, Yamagata, Japan

**Keywords:** dopamine transporter imaging, DAT-SPECT, frontotemporal dementia, frontotemporal lobar degeneration, motor neuron disease, semantic dementia

## Abstract

**Introduction:**

Frontotemporal lobar degeneration (FTLD) is a clinical syndrome with pathological heterogeneity, including Pick’s disease and trans-activating response region (TAR) DNA-binding protein with a molecular mass of 43 kDa (TDP-43) proteinopathy (FTLD-TDP). A previous study reported abnormal findings on dopamine transporter (DAT) imaging in 30% of patients with frontotemporal dementia (FTD) in FTLD. However, the previous study did not consider the pathological heterogeneity of FTD regarding the pathomechanism leading to abnormal DAT findings. Recently, abnormal DAT findings were reported in two patients with FTLD with motor neuron disease (MND), of which FTLD-TDP type B was the most common pathological presentation. This study investigated the DAT findings of patients with a final diagnosis of FTLD-MND to determine the frequency of occurrence of DAT abnormalities in FTLD-MND.

**Methods:**

Twenty patients with FTLD who underwent DAT single photon emission computed tomography (DAT-SPECT) were screened, and six patients with a final diagnosis of FTLD-MND were ultimately included. The patients’ DAT-SPECT findings were analyzed visually and quantitatively. Neuronal loss and astrogliosis in brain regions (substantia nigra, caudate, and putamen) that could possibly affect DAT findings were evaluated in the three pathologically confirmed cases.

**Result:**

All six patients with FTLD-MND showed abnormal visual DAT-SPECT findings. In addition, in a quantitative assessment, the specific binding ratio in the striatum calculated by the Southampton method was below the lower limit of the 95% prediction interval of the healthy controls by age in all the present cases. Interestingly, three of the six patients showed abnormal findings on DAT-SPECT more than half a year before the onset of MND. Neuronal loss and astrogliosis in brain regions that may affect DAT findings were observed in three pathologically confirmed cases.

**Conclusion:**

Dopamine transporter single photon emission computed tomography revealed abnormal findings in patients with FTLD-MND, which may manifest even before the onset of MND symptoms. We believe that the possibility of future development of MND should be considered if DAT-SPECT shows abnormal findings in FTLD.

## Introduction

Frontotemporal lobar degeneration (FTLD) is a clinical syndrome showing progressive behavioral or language dysfunction corresponding to the degeneration of the frontal and anterior temporal lobes and consists of frontotemporal dementia (FTD), progressive non-fluent aphasia, and semantic dementia (SD) (Neary et al., 1998). FTLD has diverse pathological conditions and is classified mainly into FTLD-tau (corticobasal degeneration, progressive supranuclear palsy, and Pick’s disease), FTLD-trans-activating response region (TAR) DNA-binding protein with a molecular mass of 43 kDa (TDP-43) (FTLD-TDP), and FTLD-fused sarcoma (FTLD-FUS) based on the protein composition of the neuronal and glial inclusions (Mackenzie et al., 2010). In addition, FTLD-TDP is categorized into types A to D based on the morphology and localization of TDP inclusions (Mackenzie et al., 2011). Such pathological diversity can make it difficult to predict the subsequent clinical course in the early stages of the disease (Yokota et al., 2021). For example, some pathologically confirmed progressive supranuclear palsy (PSP) and corticobasal degeneration (CBD) patients may have no motor symptoms in the early stage or even during their disease course (Lee et al., 2011; Sakae et al., 2019).

Dopamine transporter (DAT) single-photon emission tomography (SPECT) with ^123^I-N-omega-fluoropropyl-2-beta-carbomethoxy-3-beta (4-iodophenyl) nortropane (^123^I-FP-CIT) is a widely used diagnostic tool for patients with suspected parkinsonian syndromes such as Parkinson’s disease, PSP, and CBD as an *in vivo* marker of nigrostriatal neuron loss (Palermo and Ceravolo, 2019). In addition, abnormalities on DAT-SPECT may already appear in the prodromal stage (Hustad and Aasly, 2020). Therefore, abnormal findings of DAT-SPECT in FTLD may be a suggestive marker for predicting FTLD-tau pathology (CBD or PSP). A previous study reported abnormal findings on DAT-SPECT in 30% of patients with frontotemporal dementia in FTLD (Morgan et al., 2012), which has a heterogeneous pathological basis (Mackenzie et al., 2010). The mechanism causing the abnormal DAT-SPECT findings in FTD may vary depending on the pathological basis, but this is not discussed in the study.

Recently, abnormal DAT findings have been reported in two cases of FTLD with motor neuron disease (MND) (Kobayashi et al., 2020; Liu et al., 2020), which is mostly categorized pathologically as FTLD-TDP type B (Burrell et al., 2016). Severe degeneration of nigrostriatal neurons in FTLD with MND has been shown in autopsy series (Brun et al., 1994; Zhang et al., 2008). Therefore, DAT imaging may have detected DAT reduction, reflecting the degeneration of nigrostriatal neurons in FTLD-MND.

The purpose of this study was to investigate the DAT-SPECT findings of patients who were finally diagnosed with FTLD-MND in order to clarify whether DAT reduction in FTLD-MND was common.

## Materials and Methods

### Subjects

**FIGURE 1:**
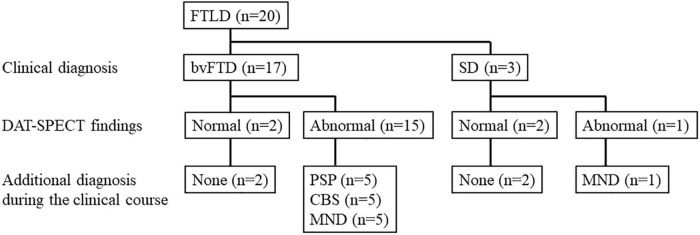
Study flow chart. bvFTD, behavioral variants of frontotemporal dementia; CBS, corticobasal syndrome; DAT-SPECT, dopamine transporter single photon emission computed tomography; FTLD, frontotemporal lobar degeneration; MND, motor neuron disease; PSP, progressive supranuclear palsy; SD, semantic dementia.

The subjects were patients with FTLD who visited the Department of Psychiatry at Yamagata University Hospital between January 2013 and March 2021. Among them, 20 patients with FTLD who underwent DAT-SPECT were screened retrospectively. Of these, 17 had behavioral variants of FTD (bvFTD) that met the diagnostic criteria of Rascovsky et al. (2011), and three had SD that met the diagnostic criteria of Neary et al. (1998). Among 17 patients, 15 (88.2%) with bvFTD showed abnormal findings on DAT-SPECT: five patients developed PSP (Höglinger et al., 2017), five developed corticobasal syndrome (Armstrong et al., 2013), and five developed MND during the clinical course. One out of the three patients (33.3%) with SD showed abnormal findings on DAT-SPECT, and this patient developed MND during the clinical course. The flow chart of participant enrollment is presented in Figure 1. This study ultimately included six patients with a final FTLD-MND diagnosis. Of the six cases, five had bvFTD, and one had SD. The bvFTD cases (case 2) included one case that we have previously reported (Kobayashi et al., 2020). Furthermore, MND was diagnosed according to the diagnostic criteria proposed by Shefner et al. (2020). Four patients out of these six underwent needle electromyography (EMG). FTLD–MND was diagnosed when both FTLD and MND diagnostic criteria were fulfilled. The Ethics Committee of Yamagata University School of Medicine approved the present study (Approval number: R1-232) and written informed consent for participation was obtained from all the patients. Written consent for the publication of the detailed case report in this manuscript was obtained from the patient and his wife.

### Dopamine Transporter Single Photon Emission Computed Tomography

^123^I-FP-CIT SPECT/computed tomography (CT) images were acquired over a period of 28 mins using a Symbia T2 with a rotating, dual-detector gamma camera (Siemens Healthineers, Erlangen, Germany) and a low-to medium-energy general-purpose collimator (Siemens Healthineers), with 360°continuous rotation (7.0 min/rotation × 4 rotations). Thereafter, X-ray CT images were acquired. Patients were placed in the supine position and intravenously injected with ^123^I-FP-CIT (167 MBq); their eyes were also closed, and their heads placed on a headrest. The acquisition condition comprised a magnification of 1.45, matrix of 128 × 128 (3.3 mm/pixel), main window of 159 ± 12.0 keV, and sub-window of 8%.

Images were reconstructed under the following conditions: three-dimensional ordered subset expectation maximization using six subsets, five iterations, and post-processing using a Gaussian filter with a full-width half maximum of 6.6 mm. CT-based attenuation correction and scatter correction using a multi-energy window and collimator broad corrections were applied.

### Dopamine Transporter Single Photon Emission Computed Tomography Analysis

**FIGURE 2:**
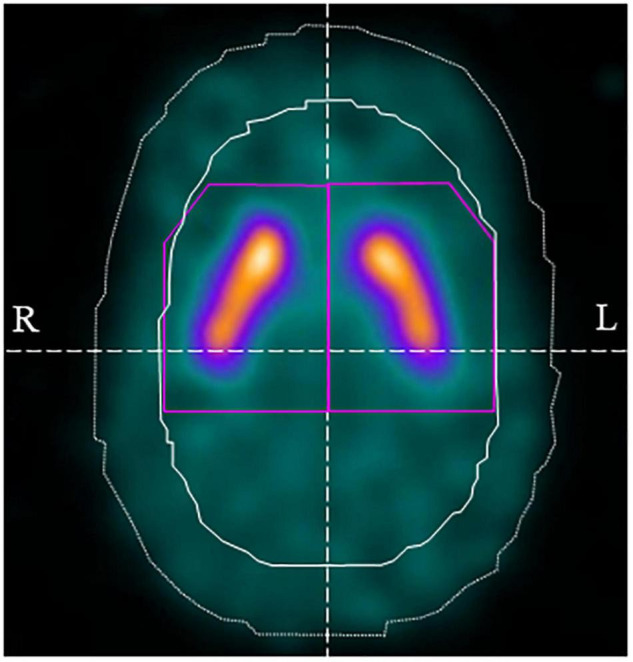
Volume of interest (VOI) setting using DaTView. The purple line shows the striatum VOI and the white solid line shows the background (non-specific) VOI.

Images were analyzed using DaTView version 5.0 (Nihon Medi-Physics, Tokyo, Japan) for automatic calculation of the calibrated specific binding ratio (SBR). Volumes of interest were set on the striatum and background regions (i.e., non-specific regions) determined using the Southampton method (Tossici-Bolt et al., 2006), and the SBR was calculated using the ratio of specific binding to non-specific accumulation of ^123^I-FP-CIT in the striatum (Tossici-Bolt et al., 2006; Figure 2). The SBR was derived from a measure of total striatal counts that takes into account the partial volume effect. The definition of the image for the analysis included transaxial slices within a “slab” approximately 44-mm thick centered on the highest striatal signal. The generous dimensions of approximately 61 mm × 48 mm ensured the inclusion of all striatal counts, including those blurred outside the actual structure because of the partial volume effect. The reference region is automatically defined from the non-specific uptake in the whole brain enclosed in the slab, with exclusion of the striatal region. The SBR was calibrated using the relationship between the measured SBR of SPECT data of a phantom and the true SBR from measurements of aliquots (Matsuda et al., 2018). In addition, the absolute value of the asymmetry index was calculated similarly to a previous study (Matsuda et al., 2018).

As a quantitative assessment, the SBR of all subjects was compared with the lower-limit SBR of the 95% lower prediction interval calculated from the database of healthy Japanese controls by age (Matsuda et al., 2018).

On visual assessment, DAT-SPECT images were classified into six types, similar to that done in recent studies using DAT-SPECT (Liu et al., 2020; Kazmi et al., 2021). That is, normal, abnormal type 1 (asymmetric accumulation, e.g., accumulation in the region of the putamen of one hemisphere is absent or greatly reduced with respect to the other), abnormal type 2 (accumulation is absent in the putamen of both hemispheres and confined to the caudate), abnormal type 3 (accumulation absent in the putamen of both hemispheres and greatly reduced in one or both caudate), and striatal loss (balanced loss to the caudate and putamen with high levels of background accumulation) (Kazmi et al., 2021). Furthermore, the pattern in which the accumulation in the caudate was significantly reduced compared with that in the putamen, as reported in a previous study (Liu et al., 2020), was defined as the caudate type.

### Pathological Methods

Neuropathological investigations were performed as previously described (Kawakatsu et al., 2017). Briefly, the brains were fixed in 10% neutral buffered formalin and cut into 0.5-cm slices. Tissue blocks were taken from approximately 30 regions, including the cerebral cortex, basal ganglia, brain stem, spinal cord, and cerebellum. The tissues were embedded in paraffin and sectioned at a thickness of 7 μm for Klüver-Barrera staining or 4 μm for hematoxylin-eosin and immunostaining. Immunohistochemistry was performed using primary antibodies against phosphorylated TDP-43 (p-TDP-43) (mouse monoclonal, clone 11-9, Cat. No. TIP-PTD-M01, pS409/410; Cosmo Bio Co., Ltd., Tokyo, Japan; 1:5,000), anti-cystatin C (rabbit monoclonal, clone EPR4413; Proteintech Group, Inc., IL, United States; 1:200), and anti-glial fibrillary acidic protein (GFAP) (mouse monoclonal, clone G-25-8-3; IBL, Gunma, Japan; 1:200). Primary antibody binding was detected using peroxidase-labeled streptavidin biotin kits (Nichirei Histofine Simple Stain Kit, MAX-PO, NICHIREI Co., Tokyo, Japan). Diaminobenzidine was used for color development, and the slides were counterstained with hematoxylin. Autoclave pretreatment was performed for antigen retrieval.

### Pathological Analysis

The subjects were three patients (bvFTD, two patients; SD, one patient) with pathologically confirmed FTLD-TDP type B. The degree of neuronal loss and astrogliosis in the substantia nigra, caudate, and putamen, which may affect DAT-SPECT (Palermo and Ceravolo, 2019), were evaluated on a 4-point scale of severe, moderate, mild, and slightest.

## Results

**FIGURE 3:**
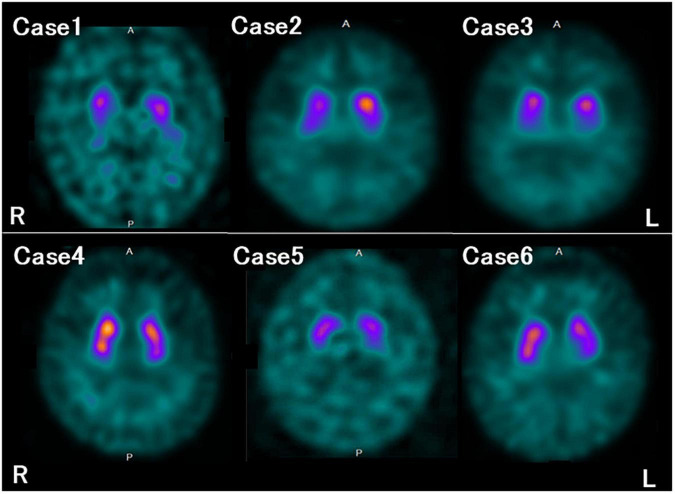
Dopamine transporter (DAT) single photon emission computed tomography (SPECT) findings in each case. All the patients in this study showed reduced DAT availability.

The characteristics and clinical data of the patients with FTLD-MND are presented in Table 1. The DAT-SPECT findings and assessments of DAT-SPECT are shown in Figure 3 and Table 2, respectively. Visual assessment using DAT-SPECT revealed abnormal findings in all cases, with abnormal type 1 in two cases, abnormal type 2 in one case, abnormal type 3 in two cases, and balanced striatal loss in one case. The average SBRs were below the lower limit of the 95% prediction interval of the healthy controls by age in all the present cases. In a survey of the interval between the time of DAT-SPECT and the onset of MND, three of the six cases showed reduced DAT from more than half a year before the onset of MND. Table 3 shows the degree of neuronal loss and astrogliosis in the brain regions (substantia nigra, caudate, and putamen) that may affect the findings of DAT-SPECT (Palermo and Ceravolo, 2019) in the three autopsied cases. Degeneration of the substantia nigra was apparent, while that of the caudate and putamen varied across the cases. These histological findings are shown in Supplementary Figure 1.

**TABLE 1:** Characteristics and clinical data of the subjects.

	Case 1	Case 2	Case 3	Case 4	Case 5	Case 6
FTLD first diagnosis	bvFTD	bvFTD	bvFTD	bvFTD	bvFTD	SD
FTLD onset age (years)	55.8	63.0	75.0	59.0	62.7	52.0
Sex	M	F	F	M	F	M
Education (years)	12	12	14	12	14	16
Age at first visit (years)	57.2	65.2	76.2	62.3	63.9	55.8
MMSE (/30)	29	18	23	27	25	27
FAB (/18)	11	3	10	15	9	11
EPMS at scan	None	None	None	None	None	None
Age when DAT-SPECT taken (years)	57.9	65.4	80.1	64.1	64.0	56.4
MND onset age (years)	59.5	65.5	79.7	67.9	64.7	56.5
Interval between DAT-SPECT and MND onset (years)	1.59	0.11	−0.39	3.80	0.69	0.10
Type of MND onset	Limb	Bulbar	Bulbar	Bulbar	Bulbar	Limb
Hyperreflexia at MND onset	None	Mild	None	None	None	None
Babinski’s sign and Hoffmann’s sign at MND onset	None	+(Babinski’s sign)	None	None	None	None
Autopsied age (years)	60.8	N/A	N/A	68.5	N/A	57.4

*bvFTD, behavioral frontotemporal dementia; SD, semantic dementia; FTLD, frontotemporal lobar degeneration; MMSE, Mini Mental State Examination; MND, motor neuron disease; FAB, frontal assessment battery; DAT-SPECT, dopamine transporter single photon emission computed tomography; M, male; F, female; EPMS, extrapyramidal motor symptoms; N/A, not available.*

**TABLE 2:** Visual and quantitative assessment for DAT-SPECT.

	Case 1	Case 2	Case 3	Case 4	Case 5	Case 6
Visual assessment of DAT-SPECT	Abnormal type 3	Abnormal type 3	Abnormal type 2	Abnormal type 1	Striatal loss	Abnormal type 1
Average SBR (right striatum/left striatum)	2.95 (2.91/2.99)	2.06 (1.96/2.15)	1.67 (1.88/1.47)	2.72 (3.54/1.90)	2.92 (3.04/2.80)	2.83 (3.22/2.44)
Lower limit SBR of the 95% PI for HC	5.41	4.98	4.07	5.04	5.04	5.53
Asymmetry index (%)	2.6	9.4	24.6	60.5	8.2	27.7

*DAT-SPECT, dopamine transporter single photon emission computed tomography; SBR, specific binding ratio; PI, prediction interval; HC, healthy controls.*

*Abnormal type 1: asymmetric accumulation, for example, accumulation in the region of the putamen of one hemisphere is absent or greatly reduced with respect to the other; abnormal type 2: accumulation is absent in the putamen of both hemispheres and confined to the caudate; abnormal type 3: accumulation absent in the putamen of both hemispheres and greatly reduced in one or both caudate, striatal loss: balanced loss to the caudate and putamen with high levels of background accumulation.*

**TABLE 3:** The degree of neuronal loss and astrogliosis in the brain regions associated with the DAT-SPECT findings.

	Case 1	Case 4	Case 6
Substantia nigra	+++	++	++
Caudate	++	+++	+
Putamen	++	+++	+

*Severe: +++, moderate: ++, mild: +.*

*DAT-SPECT, dopamine transporter shingle photon emission computed tomography.*

### Case Presentation (Case 1)

#### Clinical Course and Neuroradiological Findings

**FIGURE 4:**
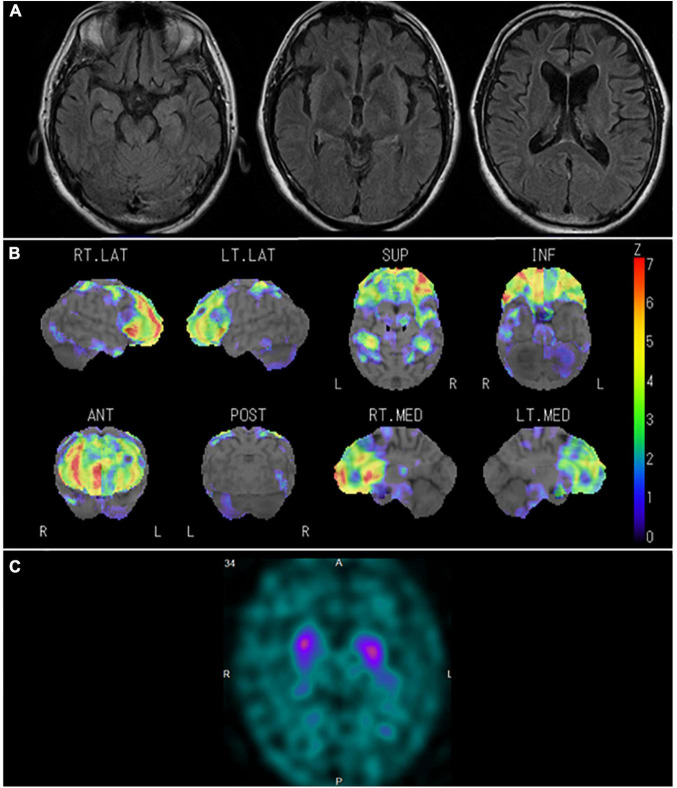
Neuroradiological findings in Case 1. **(A)** Magnetic resonance imaging shows marked bilateral frontal lobe atrophy. **(B)** Brain perfusion single photon emission computed tomography (SPECT) three-dimensional stereotactic surface projection analysis (Nihon Medi-Physics, Tokyo, Japan) shows significant bilateral frontal lobe hypoperfusion. The color scale for the Z score is shown on the right side of the figure. Presented in color if the Z-score was > 0. **(C)** Dopamine transporter (DAT) SPECT shows remarkably reduced DAT availability in the bilateral striatum.

In January 2015, the patient was constantly restless and frequented in and out of the house. In addition, he repeatedly made the same dishes and no longer cared about grooming. In June 2016, he visited our hospital for the first time. He presented a lack of insight and seriousness regarding his difficulties. Neuropsychological assessment demonstrated remarkable impairments in frontal function and attention and sparing of episodic memory and visuospatial function. Magnetic resonance imaging (MRI) (Figure 4A) of the head showed marked bilateral frontal lobe atrophy. *N*-isopropyl-*p*-[123I] iodoamphetamine SPECT revealed significant bilateral frontal lobe hypoperfusion (Figure 4B). Based on the diagnostic criteria, the patient was diagnosed with bvFTD (Rascovsky et al., 2011). He gradually presented with behaviors including overeating of sweet foods, wandering for a long time, and using stereotypical phrases (such as “all right”). In January 2017, DAT-SPECT (Figure 4C) showed remarkably reduced ^123^I-FP-CIT uptake in the bilateral striatum. In September 2018, he experienced weakness and fasciculations in his tongue and upper limbs and subsequently developed dysphagia. Needle EMG revealed neurogenic change consistent with MND. Therefore, he was diagnosed with MND. In December 2019, he died of respiratory failure.

#### Neuropathological Findings

**FIGURE 5:**
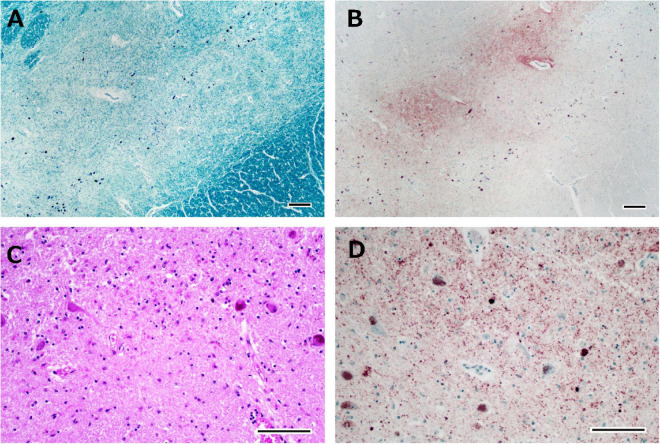
Histological observations of the substantia nigra from Case 1. Neuronal loss is observed in the intermediate portion of the substantia nigra **(A)**, where phosphorylated TDP-43-positive structures are depicted **(B)**. Astrogliosis is severe **(C)**, and phosphorylated TDP-43-positive neuronal inclusions and dot-like deposits are identified **(D)**. Klüver-Barrera staining **(A)**, immunostaining for phosphorylated TDP-43 **(B,D)**, and hematoxylin-eosin staining **(C)**. Scale bars: 200 μm **(A,B)** and 100 μm **(C,D)**.

The brain weighed 1,154 g before fixation. Macroscopic observation of the cerebrum showed moderate atrophy in the bilateral frontal lobes with a preserved primary motor cortex. The coronal sections revealed dilation of the anterior horn of the lateral ventricles with preservation of the caudate nuclei, putamen, and thalamus. Mild atrophy was noted in the medial temporal regions, including the amygdala, hippocampus, and entorhinal cortex. Depigmentation was not apparent in the substantia nigra or locus coeruleus. Microscopically, neuronal loss and astrogliosis were moderately severe in the frontal cortex, medial temporal regions, and caudate nuclei, accompanied by abundant p-TDP-43 immunoreactive neuronal inclusions, which was consistent with the findings of FTLD-TDP type B pathology. Betz cells were relatively well preserved. Neuronal loss and astrogliosis were severe in the intermediate portion of the substantia nigra, with p-TDP-43 immunoreactive dot-like deposits (Figures 5A–D), and those were moderately severe in the putamen. Bunina bodies, which were cystatin-C immunoreactive, were observed in neurons of the anterior horn of the spinal cord. Neuronal loss in the anterior horn of the spinal cord was mild.

### Case Presentation (Case 4)

#### Clinical Course and Neuroradiological Findings

**FIGURE 6:**
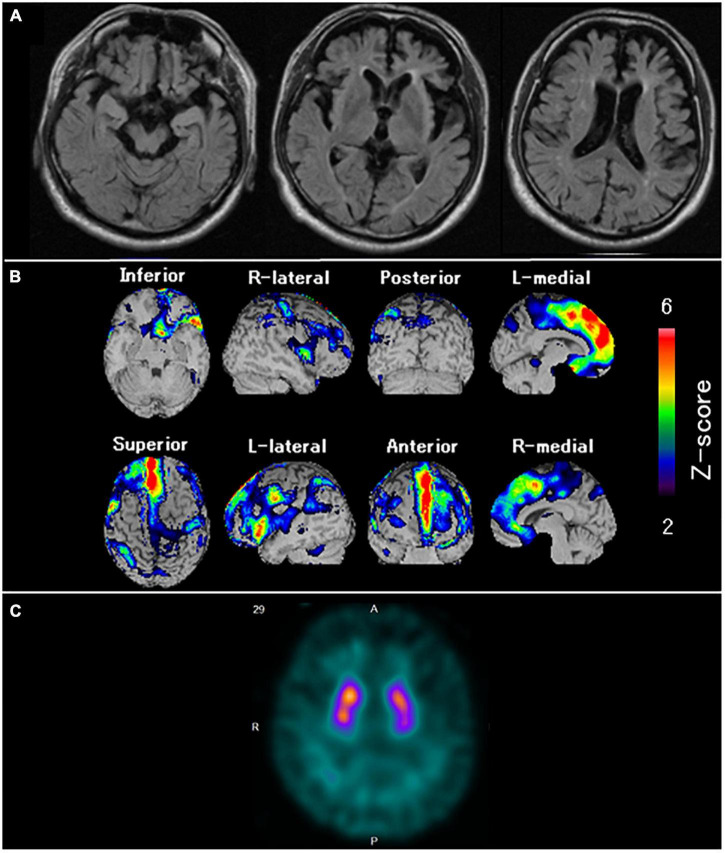
Neuroradiological findings in Case 4. **(A)** Magnetic resonance imaging shows marked bilateral frontal lobe atrophy, predominant on the left side. **(B)** Brain perfusion single photon emission computed tomography (SPECT) easy Z-score Imaging System analysis (FUJIFILM Toyama Chemical Co., Ltd., Tokyo, Japan) shows significant bilateral medial frontal lobe hypoperfusion. The color scale for the Z-score is shown on the right side of the figure. Presented in color if the Z-score was > 2. **(C)** Dopamine transporter (DAT) SPECT shows reduced DAT availability in the bilateral striatum, predominant on the left side.

In January 2011, the patient showed self-centered behavior, such as taking a break from work without permission. In May 2014, the patient visited our hospital because of irritability, disinhibition, and stereotypical behaviors such as driving the same course repeatedly every day. Neuropsychological assessment demonstrated remarkable impairment in frontal function and attention and sparing of episodic memory and visuospatial function. MRI (Figure 6A) revealed marked bilateral frontal lobe atrophy, which was predominant on the left side. Technetium-99m ethyl cysteinate dimer ([99mTc]ECD) SPECT (Figure 6B) revealed significant bilateral medial frontal lobe hypoperfusion. Based on the diagnostic criteria, the patient was diagnosed with bvFTD (Rascovsky et al., 2011). In March 2016, the patient was admitted for significant wandering behavior, sexual disinhibition, and violence. DAT-SPECT (Figure 6C) showed reduced DAT availability in the bilateral striatum, which was predominant on the left side. In June 2019, dysarthria, dysphagia, and muscle atrophy of the tongue and limbs appeared, and he was diagnosed with MND. In August 2020, the patient died of respiratory failure.

#### Neuropathological Findings

**FIGURE 7:**
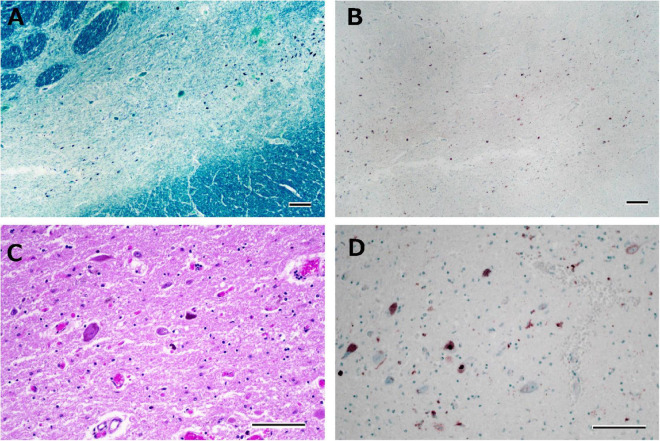
Histological observations of the substantia nigra from Case 4. Neuronal loss is observed in the intermediate portion of the substantia nigra **(A)**, where phosphorylated TDP-43-positive structures are depicted **(B)**. Astrogliosis is moderately severe **(C)**, and phosphorylated TDP-43-positive neuronal inclusions and dot-like deposits are identified **(D)**. Klüver-Barrera staining **(A)**, immunostaining for phosphorylated TDP-43 **(B,D)**, and hematoxylin-eosin staining **(C)**. Scale bars: 200 μm **(A,B)** and 100 μm **(C,D)**.

The brain weighed 1,000 g before fixation. Macroscopic observation of the cerebrum revealed moderate atrophy in the bilateral frontal lobes with preserved primary motor cortices. The coronal sections showed atrophy of the frontal cortices with preservation of the hippocampus, amygdala, and temporal cortices. The caudate nuclei, putamen, and thalamus were also intact. Depigmentation was not apparent in the substantia nigra or locus coeruleus. Microscopically, neuronal loss and astrogliosis were severe in the frontal cortices, caudate nuclei, and putamen. Many p-TDP-43 immunoreactive neuronal inclusions, which were suggestive of FTLD-TDP type B pathology, were observed throughout the frontal and frontal cortices, hippocampus, caudate nuclei, and putamen. Mild neuronal loss of Betz cells was observed. Neuronal loss and astrogliosis were moderately severe in the intermediate portion of the substantia nigra, with p-TDP-43 immunoreactive dot-like deposits (Figures 7A–D). Bunina bodies, which were cystatin-C immunoreactive, were observed in neurons of the anterior horn of the spinal cord. Neuronal loss in the anterior horn of the spinal cord was moderate.

### Case Presentation (Case 6)

#### Clinical Course and Neuroradiological Findings

**FIGURE 8:**
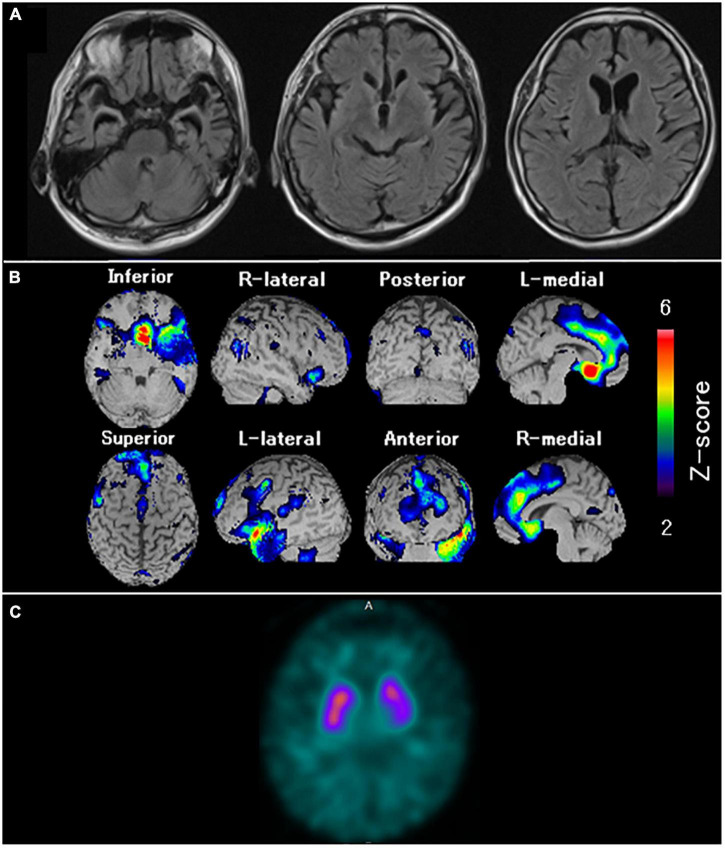
Neuroradiological findings in Case 6. **(A)** Magnetic resonance imaging shows marked bilateral anterior temporal lobe atrophy, predominant on the left side and the orbitofrontal cortex. **(B)** Brain perfusion single photon emission computed tomography (SPECT) easy Z-score Imaging System analysis (FUJIFILM Toyama Chemical Co., Ltd., Tokyo, Japan) shows significant hypoperfusion in the bilateral anterior temporal lobes, predominantly in the left side, the orbitofrontal cortex, and the medial frontal lobe. The color scale for the Z-score is shown on the right side of the figure. Presented in color if the Z-score was > 2. **(C)** Dopamine transporter (DAT) SPECT shows reduced DAT availability in the bilateral striatum, predominant on the left side.

In January 2011, the patient lost the ability to articulate names of objects and employees of the restaurant he managed. In October 2014, he received an initial examination at our hospital after showing symptoms such as an inability to understand the meaning of food names. Although his spontaneous speech was fluent, word-finding difficulties were evident. Neuropsychological assessment such as the western aphasia battery and frontal assessment battery confirmed impaired naming, impaired word comprehension, surface dyslexia, prosopagnosia, and frontal lobe disorder. MRI (Figure 8A) showed evident atrophy in the bilateral anterior temporal lobes predominant on the left side, the basal surface of the temporal lobe, and the orbitofrontal cortex. [99mTc]ECD SPECT (Figure 8B) revealed bilateral hypoperfusion in the anterior temporal lobes, which was predominant in the left lobe, the orbitofrontal cortex, and the medial frontal lobe. He was diagnosed with SD based on the diagnostic criteria of Neary et al. (1998). In July 2015, DAT-SPECT (Figure 8C) showed reduced ^123^I-FP-CIT uptake in the bilateral striatum, predominant on the left side. In August 2015, he presented with dorsal interosseous muscle atrophy of both hands and fasciculation in the bilateral upper limbs. Needle EMG revealed neurogenic change consistent with MND. Therefore, he was diagnosed with MND. In March 2016, dysphagia developed, and 3 months after the onset of dysphagia (June 2016), he was discovered in his home in a state of cardiopulmonary arrest and death from respiratory failure was confirmed at our hospital.

#### Neuropathological Findings

**FIGURE 9:**
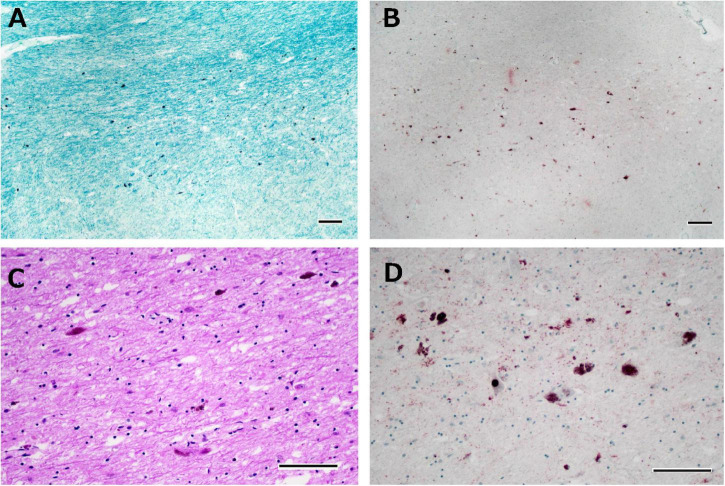
Histological observations of the substantia nigra from Case 6. Neuronal loss is observed in the intermediate portion of the substantia nigra **(A)**, where phosphorylated TDP-43-positive structures are depicted **(B)**. Astrogliosis is moderately severe **(C)**, and phosphorylated TDP-43-positive neuronal inclusions and dot-like deposits are identified **(D)**. Klüver-Barrera staining **(A)**, immunostaining for phosphorylated TDP-43 **(B,D)**, and hematoxylin-eosin staining **(C)**. Scale bars: 200 μm **(A,B)** and 100 μm **(C,D)**.

The brain weighed 1,270 g before fixation. Macroscopic observation of the cerebrum revealed moderate atrophy in the bilateral anterior temporal lobes with preserved primary motor cortices. The coronal sections showed moderate atrophy in the medial temporal regions, including the amygdala, hippocampus, and entorhinal cortices. Mild atrophy was noted in the bilateral frontal lobes. The caudate nuclei, putamen, and thalamus were intact. Depigmentation was not apparent in the substantia nigra or locus coeruleus. Microscopically, neuronal loss and astrogliosis were moderately severe in the medial temporal regions. Several p-TDP-43 immunoreactive neuronal inclusions were observed in the dentate granule cells of the hippocampus, temporal cortices, caudate nuclei, and putamen, which were suggestive of FTLD-TDP type B. Betz cells were relatively preserved. Neuronal loss and astrogliosis were moderately severe in the intermediate portion of the substantia nigra, with p-TDP-43 immunoreactive dot-like deposits (Figures 9A–D), whereas neuronal loss and astrogliosis were mild in the caudate and putamen. Bunina bodies, which were cystatin-C immunoreactive, were observed in neurons of the anterior horn of the spinal cord. Neuronal loss in the anterior horn of the spinal cord was moderate.

## Discussion

In this study, all the patients with FTLD-MND showed reduced DAT on DAT-SPECT, suggesting that FTLD-MND cases are prone to DAT reduction. In a previous study of DAT-SPECT in bvFTD, 4 of 12 cases had abnormal findings, but those with abnormal findings presented with parkinsonism, hallucinations, or non-fluent speech (Morgan et al., 2012). Therefore, some of these cases may include PSP, CBD, and FTD with parkinsonism linked to chromosome-17 (FTDP-17), which have similar clinical features (Park and Chung, 2013; Shinagawa et al., 2014) and are likely to have reduced DAT (Park and Chung, 2013; Brooks, 2016; Takeshige et al., 2018; Wu et al., 2018). That is, the pathological heterogeneity presenting with bvFTD may be related to the presence or absence of abnormal findings on DAT imaging. Considering a previous study and our results, if FTLD cases had decreased DAT on DAT imaging, it may have been necessary to consider FTLD-MND in addition to PSP, CBD (Brooks, 2016), and FTLD-17 (Takeshige et al., 2018; Wu et al., 2018). In addition, DAT had already been reduced in 3 of the present 6 cases more than half a year before the onset of MND symptoms. In other words, reduced DAT findings in FTLD may suggest a subsequent onset of MND. Similar pre-symptomatic DAT reduction findings have also been reported in the parkinsonism of FTDP-17 (Wu et al., 2018).

FTLD-MND has been reported to cause severe neuronal loss in the substantia nigra in pathological studies (Zhang et al., 2008; The Lund and Manchester groups. Clinical and neuropathological criteria for frontotemporal dementia, 1994). Even in FTLD-MND, degeneration of the substantia nigra may affect DAT reduction because DAT-SPECT is reduced, reflecting neuronal loss of the dopaminergic nigrostriatal neurons in Parkinson’s disease (Palermo and Ceravolo, 2019). Abnormal findings on DAT-SPECT have been reported in some but not all the patients with sporadic amyotrophic lateral sclerosis (ALS) (Borasio et al., 1998), which has the same pathological basis as FTLD-MND. Previous studies have shown that ALS can be divided into two types: one in which the TDP-43 pathology spreads to the frontal, temporal, and substantia nigra, and the other that has no spreading (Nishihira et al., 2008). ALS, which has a wide distribution of TDP-43 pathology, has apparent neuronal loss in the substantia nigra and is closely associated with dementia (i.e., FTLD-MND) (Nishihira et al., 2008). Therefore, the non-constant results of DAT-SPECT findings in ALS (Borasio et al., 1998) may depend on the difference in the spread of the distribution of TDP-43 pathology in ALS. Because FTLD-MND has more severe nigrostriatal neuronal loss than ALS (Nishihira et al., 2008; Zhang et al., 2008), FTLD-MND might be more likely to have abnormal findings on DAT-SPECT than ALS.

Among the subtypes of FTLD-TDP, most cases with FTLD-MND are pathologically FTLD-TDP type B (Burrell et al., 2016). Yokota et al. (2009) compared neuronal loss in the substantia nigra, caudate, and putamen between Pick’s disease and FTLD-TDP, including FTLD-TDP type B. They revealed that FTLD-TDP was characterized by more severe neuronal loss in these regions than Pick’s disease (Yokota et al., 2009). In addition, the same researchers showed the preservation of nigral neurons in Pick’s disease (Yokota et al., 2002). Based on these findings (Yokota et al., 2002, 2009), Pick’s disease may be the most unlikely abnormal finding in DAT imaging among patients with FTLD. However, it is not clear whether there is a difference in neuronal loss in the dopaminergic nigrostriatal neurons depending on the subtype of FTLD-TDP (type A–D). In the future, radiological-pathological correlation studies are needed for each pathological subtype of FTLD, including FTLD-TDP.

Recently, Liu et al. (2020) reported interesting DAT imaging findings in FTLD-MND. The patient described did not exhibit apparent bvFTD symptoms, but the identification of TANK-binding kinase 1 mutations led to the diagnosis of FTLD-MND. DAT availability assessed by 11C-N-2-carbomethoxy-3-(4-fluorophenyl)-tropane positron emission tomography was decreased in the striatum, especially in the caudate. Based on this finding, the authors suggested that reduced DAT binding in the caudate may contribute to the differentiation of FTLD-MND from other neurodegenerative disorders. In contrast, all of our cases with typical symptoms of FTLD did not exhibit a decrease in caudate-predominant DAT availability, as reported by Liu et al. (2020). Since Liu et al.’s (2020) case did not exhibit typical bvFTD symptoms, it is possible that the distribution of TDP-43 pathology did not spread to the frontal lobes. The types of disease in which TDP-43 pathology is not widely distributed in the brain, including the frontal lobe, have been shown to cause inconspicuous neuronal loss in the substantia nigra (Nishihira et al., 2008). In addition, a recent report suggested that the caudate is involved in the early-phase symptoms of FTLD and ALS (Sobue et al., 2018). Considering these reports, DAT-SPECT in Liu et al.’s case (2020) may have been unaffected by neuronal loss in the substantia nigra and may have reduced caudate-predominant DAT availability, reflecting degeneration of the caudate. In this study, not only the substantia nigra but also the caudate and putamen were pathologically evaluated, and neuronal loss and astrogliosis were observed in these areas. Therefore, FTLD-MND may be more likely to show DAT reduction on DAT-SPECT, reflecting neuronal loss in these areas. In addition, differences in the distribution of neuronal loss in these areas may result in diverse striatal DAT images. In fact, the visual assessment of DAT-SPECT in this study was inconsistent in the regions of DAT reduction.

This study has several limitations. First, it was a retrospective study with a small sample of subjects evaluated at only one medical institution. Therefore, our results should be validated in larger and multicenter prospective studies. Second, this study does not compare DAT-SPECT findings in FTLD other than FTLD-MND with DAT-SPECT findings in FTLD-MND. This is because in our case series of FTLD that underwent DAT-SPECT, there were many patients who developed PSP and corticobasal syndrome during the clinical course. In FTLD, it has been pointed out that the deviation of the proportions of clinical diagnoses has a large effect on the survey results (Yokota et al., 2021). Therefore, further studies on DAT-SPECT are needed for each pathological subtype of FTLD. Third, autopsies were not performed in all cases. Therefore, we have not investigated the relationship between the difference in the distribution of degeneration in the substantia nigra, caudate, and putamen and DAT-SPECT findings. Fourth, we could not analyze regional quantitative values of the striatum because we used DaTView for analyzing DAT-SPECT in this study. An analysis that can calculate regional quantitative values such as DaTQUANT (GE. Healthcare, Little Chalfont, United Kingdom) may provide valuable information, including the influence of the disconnection of the striatum from the frontostriatal circuits associated with frontal lobe atrophy (Bertoux et al., 2015). Fifth, this study did not consider the influence of laterality on brain atrophy. One patient with bvFTD (Case 4) and one patient with SD (Case 6) showed decreased DAT accumulation in the ipsilateral striatum, corresponding to the laterality of atrophy. Future DAT imaging studies of FTLD are warranted that consider the laterality of brain atrophy. Sixth, this study did not examine the association between DAT abnormalities and the severity of MND or the MND onset type (i.e., bulbar or limb onset). A previous DAT-SPECT study involving patients with ALS did not observe any association between striatal DAT availability and the duration of illness or the type of MND onset (Borasio et al., 1998). A previous study on FTD-MND suggested that bulbar onset MND was more frequent than limb onset MND (Mitsuyama and Inoue, 2009); all cases in our study were also of bulbar onset MND, except for one patient with bvFTD (Case 1) and another patient with SD (Case 6). Further investigation is needed to determine whether FTD-MND bears similarities to ALS with respect to the association between DAT abnormalities and the severity of MND or the MND onset type. Incidentally, although our cases did not exhibit it, some cases of ALS exhibit parkinsonism (Calvo et al., 2019). Therefore, the relationship between presence or absence of parkinsonism and DAT abnormalities also needs to be evaluated. Finally, we did not perform a genetic analysis.

In conclusion, DAT-SPECT revealed abnormal findings in cases of FTLD-MND, which may be present even before the onset of MND symptoms, unlike previously reported abnormal DAT findings concerning the presence of subtle MND signs (Kobayashi et al., 2020). We suggest that the possibility of future MND development may be considered when DAT-SPECT shows abnormal findings in FTLD. In the future, comparative studies using transcranial magnetic stimulation and other modalities such as diffusion tensor imaging (Hübers et al., 2021) that can detect degenerative changes at an early stage of MND are also needed.

## Data Availability Statement

The original contributions presented in the study are included in the article/Supplementary Material, further inquiries can be directed to the corresponding author.

## Ethics Statement

The studies involving human participants were reviewed and approved by the Ethics Committee of Yamagata University School of Medicine. The patients/participants provided their written informed consent to participate in this study.

## Author Contributions

RK conceptualized the study, conducted neuroradiological and neuropathological examinations, analyzed the data, and drafted the manuscript. SK conducted the neuropathological examinations and drafted the manuscript. MO and MK analyzed the neuroradiological examinations and drafted the manuscript. DM analyzed the clinical data and revised the manuscript. KO encouraged the study and revised the manuscript accordingly. All authors have read and approved the final version of this manuscript.

## Conflict of Interest

The authors declare that the research was conducted in the absence of any commercial or financial relationships that could be construed as a potential conflict of interest.

## Publisher’s Note

All claims expressed in this article are solely those of the authors and do not necessarily represent those of their affiliated organizations, or those of the publisher, the editors and the reviewers. Any product that may be evaluated in this article, or claim that may be made by its manufacturer, is not guaranteed or endorsed by the publisher.

## Supplementary Material

Supplementary Figure 1 Histological observations of the substantia nigra, caudate and putamen in three autopsied cases. Neuronal loss is observed in the medial portion of the substantia nigra **(A–C)**, where astrogliosis is depicted **(D–F)**. Neuronal loss and astrogliosis are also evident in the caudate **(G–I)** and putamen **(J–L)**. Klüver-Barrera staining **(A–C)** and hematoxylin-eosin staining **(D–L)**. Scale bars: 200 μm **(A–C)** and 100 μm **(D–L)**.
