# Supplementary figures and images for: Dopamine Transporter Imaging for Frontotemporal Lobar Degeneration With Motor Neuron Disease
Source: Front Neurosci. 2022 Feb 25;16:755211. doi: 10.3389/fnins.2022.755211 (PMC8914109; doi:10.3389/fnins.2022.755211)

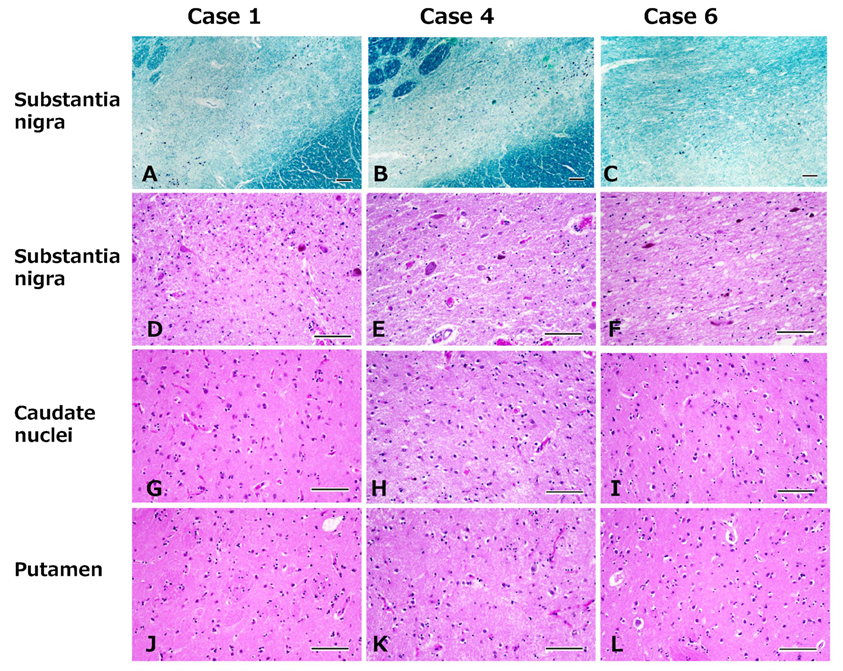

Supplement: Supplementary Figure 1 — Histological observations of the substantia nigra, caudate and putamen in three autopsied cases. Neuronal loss is observed in the medial portion of the substantia nigra (A–C), where astrogliosis is depicted (D–F). Neuronal loss and astrogliosis are also evident in the caudate (G–I) and putamen (J–L). Klüver-Barrera staining (A–C) and hematoxylin-eosin staining (D–L). Scale bars: 200 μm (A–C) and 100 μm (D–L). [file Image_1.TIF]
